# Functional Characterization of an Odorant Receptor Expressed in Newly Hatched Larvae of Fall Armyworm *Spodoptera frugiperda*

**DOI:** 10.3390/insects15080564

**Published:** 2024-07-26

**Authors:** Zhiqiang Wang, Xiaoqing Wang, Weihao Liu, Run Chen, Yang Liu

**Affiliations:** 1 State Key Laboratory for Biology of Plant Diseases and Insect Pests, Institute of Plant Protection, Chinese Academy of Agricultural Sciences, Beijing 100193, China; 82101212327@caas.cn (Z.W.); 18511602133@163.com (X.W.); liuweihao2021@163.com (W.L.); 82101212319@caas.cn (R.C.); 2 School of Tropical Agriculture and Forestry, Hainan University, Haikou 570228, China

**Keywords:** *Spodoptera frugiperda*, odorant receptor, plant volatiles, newly hatched larvae, behavior

## Abstract

**Simple Summary:**

Odorant receptors (ORs) are essential to insects’ highly efficient and sensitive olfactory system. ORs’ stage-specific expression and precise functionality significantly influence insect behavior exhibited during particular developmental stages. Despite larvae possessing a relatively simplistic olfactory system with fewer expressed ORs than adult moths, larvae effectively employ ORs to navigate larval-specific survival challenges. This study investigated the OR SfruOR40 in newly hatched larvae of *Spodoptera frugiperda*, an invasive agricultural pest also known as the fall armyworm. Subsequent functional characterization indicated that SfruOR40 facilitates the detection of host plant volatiles. Our findings enhance understanding of the olfactory perception mechanisms in *S. frugiperda* and underscore the significance of OR expression in newly hatched larvae.

**Abstract:**

In the past decade, *Spodoptera frugiperda* has emerged as a significant invasive pest globally, posing a serious threat to agriculture due to its broad diet, migratory behavior, and ability to cause extensive plant damage. While extensive research has focused on the olfactory capabilities of adult *S. frugiperda*, understanding of the olfactory process in larvae remains limited, despite larvae playing a crucial role in crop damage. To address this gap, we identified an odorant receptor (OR), SfruOR40, expressed in the first-instar larvae through phylogenetic analysis. Using quantitative real-time PCR, we compared SfruOR40 expression levels in larvae and adults. We then characterized the function of SfruOR40 against 67 compounds using the *Xenopus* oocyte expression system and found that SfruOR40 responded to three plant volatiles. Further, behavioral experiments revealed a larval attraction to (−)-*trans*-Caryophyllene oxide. This study elucidates SfruOR40’s role in the olfactory recognition of newly hatched *S. frugiperda* larvae, expanding our knowledge of such mechanisms in Noctuid moths. Furthermore, it highlights the potential of plant-derived natural products for biological pest control from a behavioral ecology perspective.

## 1. Introduction

Olfaction plays a crucial role in the behaviors of insects, ranging from influencing feeding behaviors to sex pheromones sensing in mating [1,2]. Antennae serve as the primary olfactory organs in insects by facilitating chemical signal communication between the insect and the external environment. Odor recognition is a complex process that requires coordination between many structures and proteins, which requires further research to be fully understood. In simple terms, odor recognition begins when volatile compound molecules from the habitat infiltrate lymphatic fluid through the polar pores on the cuticle of the antennal sensilla. The volatile compound molecules bind to binding proteins and are transported to olfactory receptor neurons housed within the olfactory sensilla. Odorant receptors (ORs) on the dendrite membranes of neurons are activated by the binding event and open ligand-gated ion channels, and thus the chemical signals are converted into electrical impulses [3].

Moths, belonging to the order Lepidoptera, constitute an economically important group of insects. Larvae are the primary feeding stage while adult moths engage in activities such as mating and nocturnal ovipositing. For both the larval and adult stages, olfaction plays a crucial role in guiding behaviors [4]. Using a variety of omics analyses, such as genomic analysis and transcriptome analysis, a large number of OR sequences have been identified in Noctuidae including *Heliothis virescens* [5,6], *Spodoptera littoralis* [7], *Helicoverpa armigera* [8,9], and *Helicoverpa assulta* [8]. The available omics datasets have aided in the functional characterization of ORs across species, but studies of olfaction in moths have been largely limited to the adult stage and have focused on understanding mating behaviors, such as how ORs detect sex pheromones [10,11,12]. In addition to understanding mating behaviors, research has focused on olfactory recognition circuits that are either central or peripheral in adults [13,14,15,16], and also explored evolutionary events in olfactory genes, such as interspecies functional conservation of key genes to adapt to habitat [17,18,19]. In contrast, there are only a few reports describing chemosensory genes expressed at the larval stage [20,21,22]; thus, the molecular and physiological mechanisms underlying larval olfactory behavior remain unclear. Although the larval stage is a major cause of plant injury causing significant economic burden [20], there is a lack of understanding of the molecular mechanisms of larval olfaction and the resulting olfaction-driven behavior.

Adult moths participate in mating, spawning, and other key behaviors for population reproduction [10,14,23], so researchers often prioritize the study of adults in pest control. While targeting adults to effectively manage and control pest populations is valuable, the larvae stage should not be overlooked, as it constitutes the primary period of harm to crops. Lepidopteran larvae possess a relatively simple olfactory system, with the antennae and maxillae serving as the principal chemosensory organs situated on the caterpillar’s head [24]. During feeding, the larvae search for, locate, and recognize the host plant mainly through the ORs on the antenna and the gustatory receptors on the mouth, which give the larvae the option to eat the plant [4]. Larvae express less OR than adults, so to compensate, larvae have an improved ability to search for chemical signals through combinatorial coding, that is, an odorant can activate more than one OR [20]. Newly hatched larvae have a particularly unique and challenging situation because they must establish themselves on a food plant [25]. Previous research has demonstrated that there are changes in the ability to detect odors and in the peripheral olfactory system during the development of larvae. Specifically, the OR expression of the first hatched larvae was higher than that of subsequent instars, meaning there is plasticity of olfactory perception in the larval stage itself [22]. Whether this interesting phenomenon is widespread in moths has not yet been explored. Research on the olfactory molecular mechanisms of larvae, particularly newly hatched larvae, are complimentary to efforts targeting control of adult moths. Moreover, it advances our mechanistic understanding of the olfactory sense in moths.

The fall armyworm, *Spodoptera frugiperda* (Lepidoptera, Noctuidae), is a well-documented agricultural pest with a native range spanning North and South America. However, it has recently emerged as a significant invasive pest globally, particularly in the past decade [26,27]. It invaded China in December 2018 [28,29,30]. Since the spread of this polyphagous pest, numerous staple crops, including *Oryza sativa* (rice), *Triticum aestivum* (wheat), and *Zea maize* (corn), have been infested, which poses a significant threat to food security. In China, the total area of cropland infested with them exceeded 1.125 million hectares in 2019 and 1.278 million hectares in 2020 [31]. Therefore, the invasion of *S. frugiperda* has garnered widespread attention from governments and researchers.

To handle the challenge of invasive pests, multiple aspects of monitoring and integrated management strategies have been conducted [32,33,34]. Biological control methods, such as the rearing and release of parasitic wasps and the application of insect pathogens or viruses products, reduced the use of chemical pesticides [35,36]. It has been observed that sexual communication can evolve rapidly in a new environment to reduce communication interference from endemic species [37]. Therefore, region-specific pheromone lures are more effective at attracting invasive *S. frugiperda*, meaning that the targeted trap design will be more convenient for monitoring and managing them. To achieve this purpose of designing targeted pheromone lures, many studies of the ORs in adult *S. frugiperda* have been conducted, such as the function of the sex pheromone receptor and evolutionary analysis in related species [38,39,40,41]. Nevertheless, research on larval ORs has been primarily confined to the identification and analysis of expression patterns [42]. Here, we cloned the OR *SfruOR40* from newly hatched *S. frugiperda* larvae based on published work in *S. littoralis* [22]. Quantitative expression analysis of SfruOR40 using quantitative real-time PCR (qPCR) confirmed this OR expression is in not only adults, but also in newly hatched larvae. Through heterologous expression in the *Xenopus* oocyte system, coupled with two-voltage clamp electrophysiology, we characterized the function of SfruOR40 as sensing α-Humulene, (−)-*trans*-Caryophyllene, and (−)-*trans*-Caryophyllene oxide. α-Humulene and (−)-*trans*-Caryophyllene are notable because they are volatiles commonly found in the *S. frugiperda* host plants, maize and cotton. These results provide additional evidence for the possibility of plant-derived natural products for biological pest control from the behavioral ecology perspective.

## 2. Materials and Methods

### 2.1. Insect Rearing and Tissue Collection

*S. frugiperda* specimens were initially gathered from a maize field in Anhui Province, China (31°50′ N 117°0′ E), in March 2019. The insect lineage was preserved at the Institute of Plant Protection, Chinese Academy of Agricultural Sciences, Beijing, China. Larvae were reared on an artificial diet in individual glass tubes under controlled conditions: 26 ± 2 °C temperature, (60 ± 5%) relative humidity, and a 14 h light/10 h dark photoperiod. After pupation, pupae were placed in a Petri dish, which was then placed at the bottom of a metal mesh cage. The cage was covered with gauze, serving as an oviposition substrate, and cotton balls soaked in a 10% honey solution were provided as a food source for emerging adults to ensure their physiological development. The gauze containing egg masses was collected and replaced daily for subsequent experiments. Heads from newly hatched first-instar larvae (hatched within 12 h, 300 in total) and 35 pairs of antennae from the virgin male (1–3 days old) and female adults were rapidly dissected, flash-frozen in liquid nitrogen, and kept at −80 °C.

### 2.2. RNA Isolation and cDNA Synthesis

Total RNA was isolated using the RNeasy Plus Universal Mini Kit (QIAGEN, Hilden, Germany) following the manufacturer’s protocol. RNA concentration and purity were measured with a NanoDrop 2000 spectrophotometer (Thermo Fisher Scientific, Waltham, MA, USA). Using 1 μg of total RNA, cDNA was synthesized with the TransScript One-Step gDNA Removal and cDNA Synthesis SuperMix (TransGen Biotech, Beijing, China). The cDNA was then stored at −20 °C.

### 2.3. Phylogenetic Analysis

The dataset of OR sequences utilized to construct the phylogenetic tree encompassed ORs from three Noctuidae moths: *S. frugiperda* [43,44], *S. littoralis* [45,46], and *H. armigera* [17]. Amino acid sequences of these ORs were aligned using MAFFT version 7. The maximum-likelihood OR phylogeny was constructed using FastTree 2.1.8 employing the Jones–Taylor–Thornton (JTT) amino acid substitution model [47]. Node support was assessed using a Shimodaira–Hasegawa-approximate likelihood ratio test (SH-aLRT) [48]. The phylogenetic tree was visualized and tinted using FigTree 1.4.0.

### 2.4. qPCR

The expression levels of *SfruOR40* in newly hatched first-instar larval heads and adult antennae were examined using qPCR on the ABI QuantStudio 5 (ThermoFisher Scientific, Waltham, MA, USA). *SfruActin* was employed as a reference gene [39]. Gene-specific primers were designed using NCBI Primer-BLAST (https://www.ncbi.nlm.nih.gov/tools/primer-blast, accessed on 17 August 2023), and the primer sequences are presented in Table 1. Primer amplification efficiency was determined using standard curves generated from 5× serial dilutions of cDNA templates. Each qPCR reaction was conducted in triplicate for three independent biological replicates. The reaction mix containing 10 μL of 2× TransStart Green qPCR SuperMix (TransGen Biotech, Beijing, China), 1 μL of cDNA template, 0.5 μL of forward and reverse primers (10 μM), and 8 μL of RNase-free water in a total volume of 20 μL. The reactions were carried out in a 96-well plate, with the following thermal cycling conditions: an initial denaturation for 2 min at 95 °C, followed by 40 cycles of 15 s at 95 °C and 1 min at 60 °C. A melting curve was subsequently analyzed to verify the specificity of the SYBR Green PCR signal. The relative gene expression of the target gene was computed using the 2^−ΔΔCT^ method [49]. Statistical significance of variations in the relative expression level of the *SfruOR40* in the larval and adult tissue was determined using one-way ANOVA and Tukey’s test for multiple comparisons, as implemented in GraphPad Prism 6 (GraphPad Software Inc., San Diego, CA, USA).

### 2.5. OR Cloning and Sequence Analysis

The open reading frame (ORF) of SfruOR40 was cloned from larval cDNA as template utilizing gene-specific primers (Table 1). The 50 μL PCR reaction comprised 5 μL of cDNA, 25 μL of 2× PrimeSTAR HS, 2.5 μL of each primer (10 μM), and 15 μL of double-distilled water (ddH2O). The contents were combined and spun briefly in a microcentrifuge before being placed in the thermal cycler. The PCR cycling progressed as follows: 98 °C for 3 min, followed by 35 cycles of 98 °C for 10 s, 55 °C for 15 s, 72 °C for 90 s, and a final extension at 72 °C for 10 min. PCR amplification products were resolved on a 1.0% agarose gel and verified via DNA sequencing. Nucleic acid sequences were translated into amino acid sequences using the EXPASY (Expert Protein Analysis System) Translate tool (http://web.expasy.org/translate, accessed on 31 August 2023). Transmembrane domain prediction was completed using TOPCONS (https://topcons.net/, accessed on 31 August 2023).

### 2.6. Expression Vector Construction and cRNA Synthesis

The complete open reading frame (ORF) of *SfruOR40* was subcloned into the eukaryotic expression vector pT7Ts by incorporating primers containing Kozak sequences and restriction enzyme cleavage sites (See Table 1 for primer sequences). A previous paper already described documentation for the expression vector of the *SfruOrco* [38]. The pT7Ts plasmids containing target genes were completely digested using *SmaI* and then were used as a template to produce capped RNAs (cRNAs) facilitated using an mMESSAGE mMACHINE T7 kit (Ambion, Austin, TX, USA).

### 2.7. Chemicals

A total of 67 plant volatile organic compounds (VOCs) were examined (Table S1), with purity levels of ≥95% procured from Sigma-Aldrich (Saint Louis, MO, USA), Tokyo Chemical Industry Shanghai (Shanghai, China), or J&K Scientific (Beijing, China). They are plant volatiles in the living environment of moths, and have been used in olfactory recognition studies of moth adults or larvae, and most of them have been recognized by moths [17,20]. These compounds were dissolved in dimethyl sulfoxide (DMSO) as stock solutions (1 M) and kept at −20 °C. Prior to experimentation, stock solutions were diluted in 1× Ringer’s buffer (96 mM NaCl, 2 mM KCl, 5 mM MgCl_2_, 0.8 mM CaCl_2_, and 5 mM HEPES, pH 7.6) to a working concentration of 10^−4^ M. The negative control consisted of 1× Ringer’s buffer containing 0.1% DMSO.

### 2.8. Gene Expression in Xenopus Oocytes and Electrophysiological Recordings

*Xenopus* oocytes (stages V–VII) underwent microinjection with 27.6 nL of the cRNA mixture (SfruOR40:SfruOrco, 1:1, 1 ng/nL each). The microinjected oocytes were incubated in an incubation solution (96 mM NaCl, 2 mM KCl, 5 mM MgCl_2_, 0.8 mM CaCl_2_, and 5 mM HEPES, pH 7.6, 50 μg/mL tetracycline, 100 μg/mL streptomycin, and 550 μg/mL sodium pyruvate) at an incubating case under 18 °C. Following 3–4 days of incubation, the current value of each oocyte was recorded using a two-electrode voltage clamp (OC-725C oocyte clamp; Warner Instruments, Holliston, MA, USA) at −80 mV. Oocytes were stimulated by various VOC solutions diluted to 10^−4^ M throughout the recordings, with intervals between exposures to allow for the current to revert to its baseline level. The water-injected oocytes were used as a control. Data were acquired and analyzed using Digidata 1440A and pCLAMP 10.2 software (Axon Instruments Inc., Union City, CA, USA). Response spectra graphs were produced utilizing GraphPad Prism 5 (GraphPad Software, San Diego, CA, USA).

### 2.9. Behavior Assays

To test the olfactory responses of newly hatched larvae to different chemicals, two-choice behavior assays were performed following previous reports with some modifications [17,50]. Ten newly hatched first-instar larvae were positioned in the center of a plastic 6 cm diameter Petri dish. Two 1 × 1 cm filter papers containing 10 μg of each chemical diluted in hexane and solvent (hexane) were placed on opposite ends for choice behavior testing. The two-choice zones were differentiated by 2.3 cm radius half circles centered at each filter paper. The number of larvae in respective zones was documented after 30 min, and a choice index was computed as (O − C)/T, where O is the number of larvae in the odorant zone, C is the number of larvae in the solvent zone, and T is the total number of tested larvae [17,50]. The statistical significance of variations in choice indexes compared with 0 was determined using Student’s *t*-test [7].

## 3. Results

### 3.1. Identification of a Candidate OR Expressed in Newly Hatched Larvae of Noctuidae Moths Using Phylogenetic Analysis

**Figure 1:**
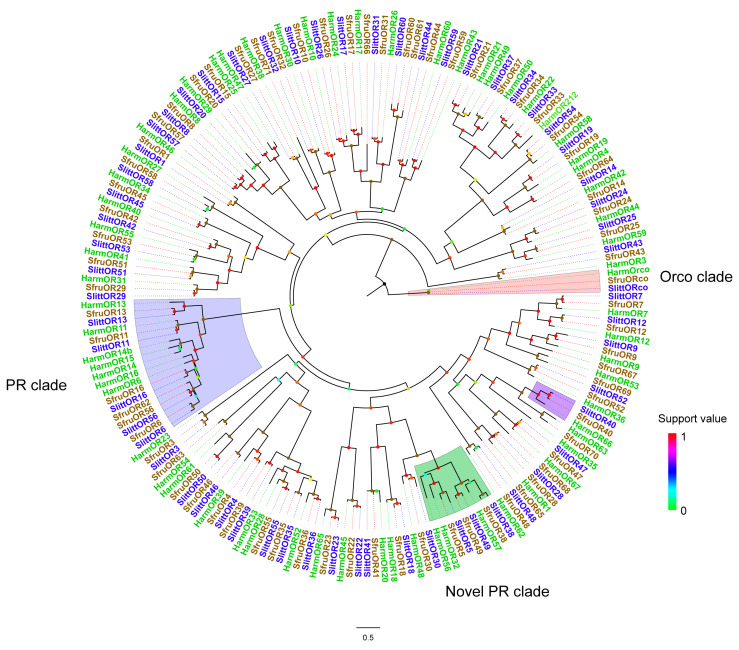
Maximum-likelihood phylogeny constructed with amino acid sequences of ORs from *S. frugiperda*, *S. littoralis*, and *H. armigera*. The Orco clade is highlighted in red, the PR clade is highlighted in blue and the novel PR clade is highlighted in green; the OR clade of interest is highlighted in purple. A total of 196 sequences were included. OR, odorant receptor; PR, pheromone receptor.

In a previous report, the ORs expressed at the larval stage were systematically studied in a noctuid moth, *S. littoralis* [22]. A special OR, SlittOR40, was found to be expressed in newly hatched larvae and could be activated by (−)-*trans*-caryophyllene and its isomer α-humulene. In that report, tuning spectra were screened against a panel of 54 volatiles [22]. Thus, we performed a phylogenetic analysis using ORs from three Noctuidae moth species to identify the candidate newly hatched larvae expressing ORs in these three moths (Figure 1). The Orco clade, PR clade, and novel PR clade of all three species are well clustered together. SfruOR40 was clustered in a well-supported clade with SlittOR40 as well as HarmOR66.

### 3.2. Gene Cloning and Sequence Analysis of Sfruor40

**Figure 2:**
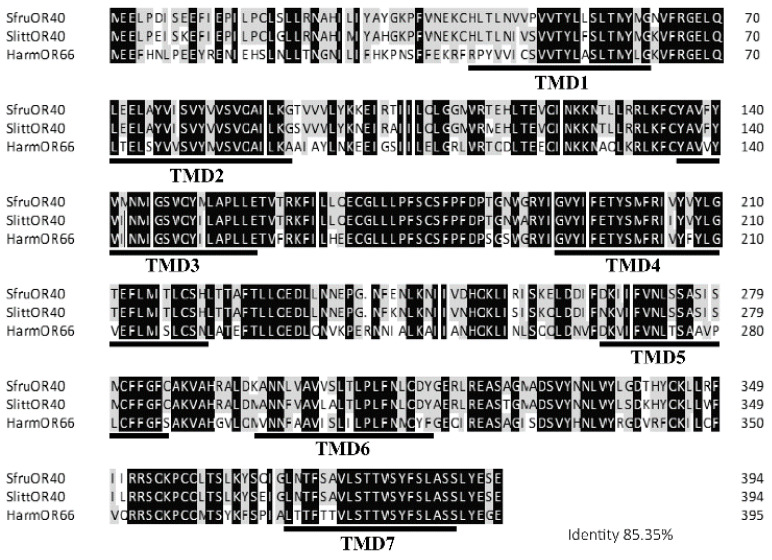
Sequence alignment and transmembrane domain predictions of SfruOR40, SlittOR40, and HarmOR66. Amino acid sequence identity is 83.35%, and is indicated as either completely identical (black) or highly identical (grey). Common TMD is underlined below. TMD, transmembrane domain.

SfruOR40 was amplified using PCR using a mixture of larvae and adult cDNA based on the reported sequence (Genbank ID: XM_035582148.2). The full-length sequence of SfruOR40, which encodes a 395 amino acid protein was acquired. A multiple sequence alignment of SfruOR40, SlittOR40, and HarmOR66 revealed a high degree of conservation among them, with 85.35% amino acid sequence identity. Prediction of the transmembrane domain (TMD) for each OR indicated the presence of a typical seven TM domains and an intracellular N-terminus (Figure 2).

### 3.3. Expression Profile of SfruOR40 in S. frugiperda

**Figure 3:**
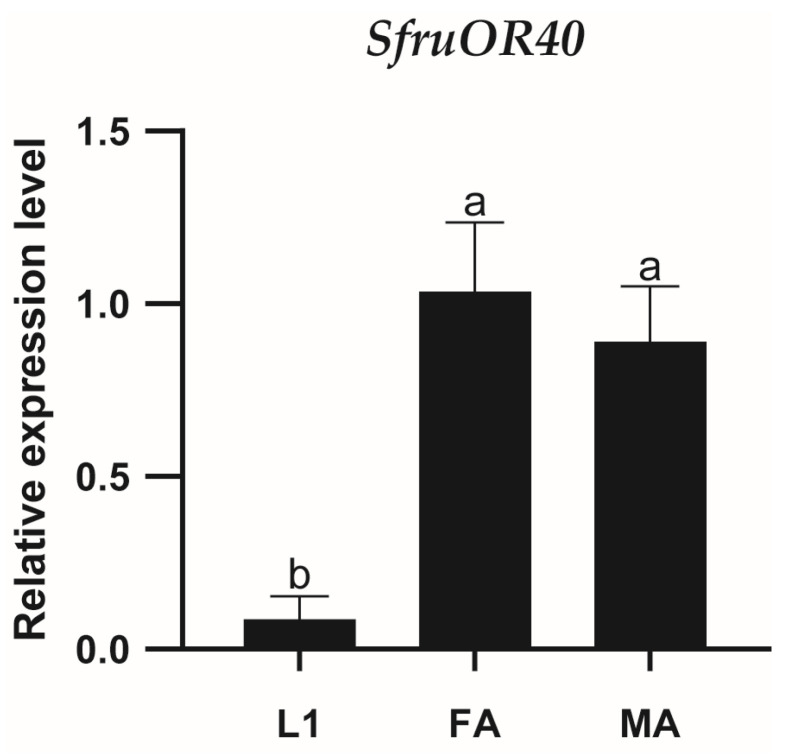
Relative expression of SfruOR40. L1, first instar larval head; FA, female adult antennae; MA, male adult antennae. Values are means ± SE. Different letters (a and b) indicate significant differences, and the same letters indicate no significant differences (one-way ANOVA and Tukey’s test for multiple comparisons, *p* < 0.05, *n* = 3).

The expression patterns of *SfruOR40* in the head of the first instar larvae, male adult antennae, and female adult antennae of *S. frugiperda* were examined using qPCR. *SfruOR40* expression in the head of the first instar larvae was identified. Higher expression levels of SfruOR40 were detected in the adult antennae compared to the larval head, with no significant difference observed between male and female antennae (Figure 3).

### 3.4. Functional Characterization of SfruOR40

**Figure 4:**
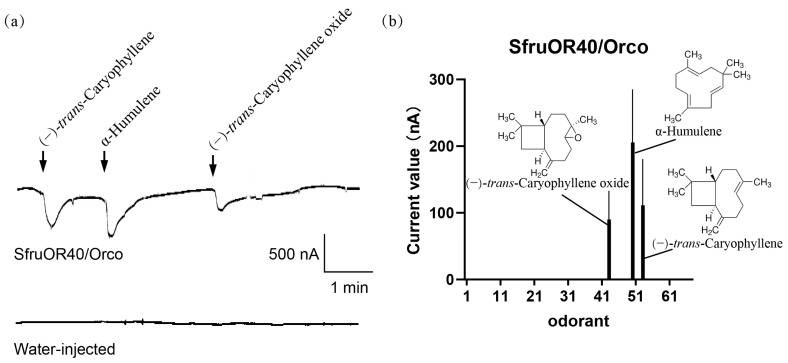
Responses of co-expressed SfruOR40/Orco in *Xenopus oocytes* to different plant volatiles. (**a**) Inward current responses to a 10^−4^ M solution of VOCs of cRNA-injected oocyte and water-injected oocyte. (**b**) Response profiles of SfruOR40/Orco *Xenopus* oocytes. Error bars indicate mean responses ± SEMs (*n* = 7).

We utilized a heterologous expression system to characterize the function of SfruOR40, which was commonly used in such functional research of odorant receptors. SfruOR40 was co-expressed with its coreceptor SfruOrco in *Xenopus* oocytes. Oocyte responses to a panel of 67 plant volatile compounds were recorded using two-electrode voltage-clamp physiological recording technique. The statistical data is measured in pCLAMP 10.2 software using cursor pairs to measure the current change before and after stimulation. Detectable current changes were recorded in oocytes expressing SfruOR40/Orco when exposed to α-Humulene, (−)-*trans*-Caryophyllene, and (−)-*trans*-Caryophyllene oxide. The mean amplitudes evoked by these compounds were 206 nA, 111 nA, and 90 nA, respectively (Figure 4, Table S2).

### 3.5. Attractiveness of Host Plant Volatiles to S. frugiperda Larvae

**Figure 5:**
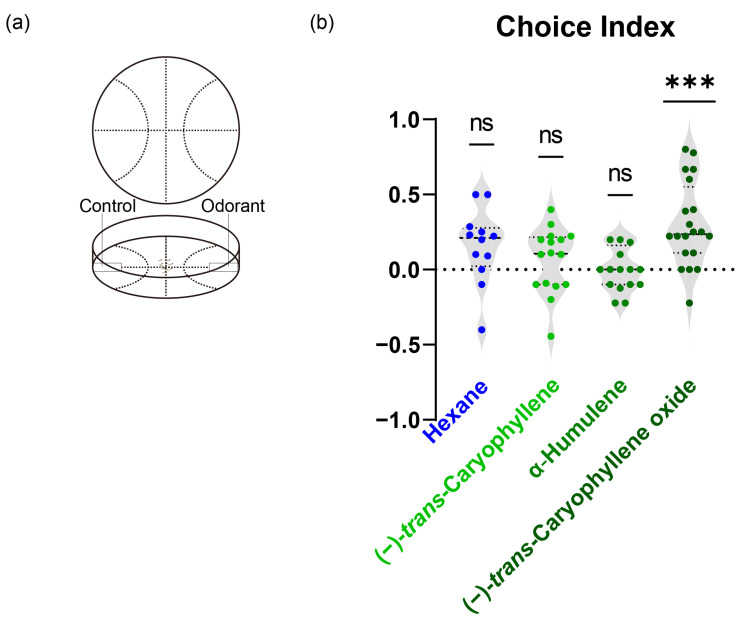
Two-choice chemotaxis behavioral assay for *S. frugiperda* larvae. (**a**) Schematic diagram of the experimental arena of larval behavioral assay. (**b**) Response profiles of first instar *S. frugiperda* larvae to three odorants: α-Humulene, (−)-*trans*-Caryophyllene, and (−)-*trans*-Caryophyllene oxide. Choice indexes significantly different from 0 are indicated by *** (*p* < 0.0001); ns, no significant difference.

To investigate if the ligands of SfruOR40 would attract or repel the first instar *S. frugiperda* larvae, we conducted a two-choice behavioral assay in a Petri dish (Figure 5a). We first ran a blank test using a two-choice behavior assay, in which a solvent (hexane) was dripped on a filter paper on both sides of the Petri dish. The first instar *S. frugiperda* larvae showed no preference between the two sides, with a choice index that was not significantly different from 0 (0 = no preference), indicating the reliability of the two-choice behavioral assay.

Subsequently, we observed the behavior of larvae to filter paper with solvent versus filter paper treated with test odorants: (−)-*trans*-Caryophyllene, α-Humulene, and (−)-*trans*-Caryophyllene oxide. In the experiments with a 10-μg dose of (−)-*trans*-Caryophyllene, first instar larvae showed no preference (mean of choice index = 0.06, *t* = 1.138, *p* = 0.273) nor for α-Humulene over solvent (10 μg: mean of choice index = 0.002, *t* = 0.05953, *p* = 0.9533). However, 10 μg of (−)-*trans*-Caryophyllene oxide induced a preference compared to control (mean of choice index = 0.30, *t* = 4.769, *p* < 0.0001) (Figure 5b).

## 4. Discussion

In this study, we focus on functional analysis of the OR SfruOR40 expressed in newly hatched *S. frugiperda* larvae. The functional exploration of uncharacterized ORs commonly begin with a phylogenetic analysis due to the shared structures and functions of homologous genes [51,52,53]. Such an approach provides advanced insights into the evolutionary relationships among these receptors. Phylogenetic analysis across three closely related moth species revealed that SfruOR40 clustered together with SlittOR40 and HarmOR66 within the same OR’s clade. Predictions of the TMDs suggest that SfruOR40 conforms to the typical features of insect ORs, displaying seven transmembrane domains with an intracellular N-terminus [3,54]. Multiple sequence alignments of three ORs from closely related species, *S. littoralis* and *H. armigera*, indicate a high sequence identity, implying that they are likely to have similar expression patterns and functions.

A study of the expression of odorant receptors in different tissues can predict the possible function of odorant receptors. The tissue expression patterns of various odorant receptors are highly variable, as has been found in *S. littoralis* [7,55], *Heliothis virescens* [56], and *Bombyx mori* [21]. ORs exhibit unique and distinct expression patterns, with some showing high or tissue-specific expression, while others display no significant difference between male and female adult antennae. Through qPCR-based expression profiling, we observed that SfruOR40 is expressed in both adult and larval stages, which aligns with SlittOR40 results in previous work [22]. This result suggests a potential function of SfruOR40 in recognizing chemicals related to individual life in adult and first instar larval stages, as is the role of SlittOR40.

We screened to identify the cognate candidate chemicals to SfruOR40 and found that it responds to (−)-*trans*-Caryophyllene, its isomer α-Humulene, and (−)-*trans*-Caryophyllene oxide. (−)-*trans*-Caryophyllene and α-Humulene are two volatile molecules that are commonly produced by plants, including host plants of *S. frugiperda*, as a reaction to herbivory [57,58]. Our result agrees with a previous study on the orthologous SlittOR40, which responds to (−)-*trans*-Caryophyllene and α-Humulene [22], further suggesting that these homologous genes may have similar or the same function. In addition, these two results were received from *Xenopus laevis* and *Drosophila melanogaster* heterogenous expression systems, respectively, which are generally consistent.

Behavioral studies have demonstrated that (−)-*trans*-Caryophyllene oxide, one of the cognate ligands of SfruOR40, has a notable impact on the choice of first instar larvae. Since larvae often possess a relatively simple olfactory system, there are fewer types of ORs expressed in larval chemosensory organs than in adults [20], and some ORs expressed in specific stages are downregulated in other stages [22]. In the case of ORs that are expressed in the larval stage, these ORs may be involved in the combinatorial coding of odor molecular signals, and their molecular ligands as chemical signals are more likely crucial for the survival of early larvae [20,22]. (−)-*trans*-Caryophyllene and α-Humulene are terpenes emitted by plants such as cotton, a preferred host of *S. frugiperda*. Interestingly, larvae showed no preference for these compounds, however, in experiments with closely related species, these molecules were able to attract *S. littoralis* larvae [22]. This previous result could be because the study added cotton leaves as background odor in the behavioral experiment, and the larvae might need to experience cotton leaf volatiles at the same time to be attracted to these compounds. However, our study did not directly confirm the clear relevancy of OR on ligands through gene knockout and behavioral experiments, which has certain shortcomings, but by performing two-electrode voltage-recording (TEVC) coupled with behavioral assays, we can say that SfruOR40 is involved in the perception of (−)-*trans*-Caryophyllene oxide by the newly hatched larvae. At least, our results regarding the larval attraction properties of (−)-*trans*-Caryophyllene oxide have the potential for the lure design of larval monitoring devices.

It has been hypothesized that increasing rates of α-Humulene signals herbivore-induced plant damage and, therefore, competition for food [58]. (−)-*trans*-Caryophyllene and α-Humulene have been associated with emitted plant volatiles that affect host plant selection for oviposition in *S. frugiperda* and other moth species [59,60,61,62]. However, the behavioral responses of larvae to (−)-*trans*-Caryophyllene and α-Humulene have not been fully determined. One study reported that the neonate larvae of *S. frugiperda* are attracted to volatile blends, including α-Humulene induced by conspecifics’ foraging [63]. The ecological relevance of such differential behavior according to species remains to be elucidated. Nonetheless, our study sheds light on the molecular mechanism behind this behavior for the first time. In the future, this may inform new invasive insect pest management strategies based on targeted interference at appropriate developmental stages for larval control from the perspective of behavioral ecology. In addition, it is worth getting insights into these questions: (1) In addition to OR40, which OR is expressed in newly hatched *S. frugiperda* larvae? (2) How about the fluctuation of transcription level from newly hatched larval until adult stage? and (3) What are the potential ecological implications of these odorant receptors?

## Figures and Tables

**Table 1:** The gene-specific primers used in this study.

Usage	Primer Name	Primer Sequences
Gene cloning	SfruOrco-F	ATGATGACCAAAGTGAAAGCCC
	SfruOrco-R	TTACTTGAGCTGCACCAACACC
	SfruOR40-F	ATGGAAGAGTTGCCTGATATTTCA
	SfruOR40-R	TCAAATTTCACTTTCGTATAGACTTGAA
Vector construction	SfruOrco-F	TCAGGGCCCgccaccATGATGACCAAAGTGAAAGCCC (*Apa* I)
	SfruOrco-R	TCAGCGGCCGCTTACTTGAGCTGCACCAACACC (*Not* I)
	SfruOR40-F	TCAGGGCCCgccaccATGGAAGAGTTGCCTGATATTTCA (*Apa* I)
	SfruOR40-R	TCAGCGGCCGCTCAAATTTCACTTTCGTATAGACTTGAA (*Not* I)
qPCR	SfruOR40-F	TCGGATTTTGTGCTAAGGTCG
	SfruOR40-R	TCTCAACCGCTCCCCGTAAT
	SfruActin-F	GGTTGGTATGGGTCAGAAGG
	SfruActin-R	AGCTCGTTGTAGAAGGTGTG

The restriction enzyme site of the prime is underlined. Kozak sequence is lowercase.

## Data Availability

All the data and resources generated for this study are included in the article.

## Supplementary Materials

The following supporting information can be downloaded at: https://www.mdpi.com/article/10.3390/insects15080564/s1, Table S1: Test compounds in this study. Table S2: Raw data of functional characterization of SfruOR40.

## References

[1] Hildebrand J.G. (1995). Analysis of Chemical Signals by Nervous Systems. Proc. Natl. Acad. Sci. USA.

[2] Masson C., Mustaparta H. (1990). Chemical Information Processing in the Olfactory System of Insects. Physiol. Rev..

[3] Leal W.S. (2013). Odorant Reception in Insects: Roles of Receptors, Binding Proteins, and Degrading Enzymes. Annu. Rev. Entomol..

[4] Kurtovic A., Widmer A., Dickson B.J. (2007). A Single Class of Olfactory Neurons Mediates Behavioural Responses to a *Drosophila* Sex Pheromone. Nature.

[5] Krieger J., Grosse-Wilde E., Gohl T., Dewer Y.M.E., Raming K., Breer H. (2004). Genes Encoding Candidate Pheromone Receptors in a Moth (*Heliothis virescens*). Proc. Natl. Acad. Sci. USA.

[6] Vogel H., Heidel A.J., Heckel D.G., Groot A.T. (2010). Transcriptome Analysis of the Sex Pheromone Gland of the Noctuid Moth *Heliothis virescens*. BMC Genom..

[7] Poivet E., Gallot A., Montagné N., Glaser N., Legeai F., Jacquin-Joly E. (2013). A Comparison of the Olfactory Gene Repertoires of Adults and Larvae in the Noctuid Moth *Spodoptera littoralis*. PLoS ONE.

[8] Zhang J., Wang B., Dong S.L., Cao D.P., Dong J.F., Walker W.B., Liu Y., Wang G.R. (2015). Antennal Transcriptome Analysis and Comparison of Chemosensory Gene Families in Two Closely Related Noctuidae Moths, *Helicoverpa armigera* and *H. assulta*. PLoS ONE.

[9] Liu Y., Gu S., Zhang Y., Guo Y., Wang G. (2012). Candidate Olfaction Genes Identified within the *Helicoverpa armigera* Antennal Transcriptome. PLoS ONE.

[10] Liu Y., Heath J.J., Zhang S., Wijk M.V., Wang G.R., Buellesbach J., Wada-Katsumata A., Groot A.T., Schal C. (2023). A Mosaic of Endogenous and Plant-Derived Courtship Signals in Moths. Curr. Biol..

[11] Cao S., Huang T.Y., Shen J., Liu Y., Wang G.R. (2020). An Orphan Pheromone Receptor Affects the Mating Behavior of *Helicoverpa armigera*. Front. Physiol..

[12] Cao S., Sun D.D., Liu Y., Yang Q., Wang G.R. (2023). Mutagenesis of Odorant Coreceptor Orco Reveals the Distinct Role of Olfaction between Sexes in *Spodoptera frugiperda*. J. Integr. Agric..

[13] Corcoran J.A., Jordan M.D., Thrimawithana A.H., Crowhurst R.N., Newcomb R.D. (2015). The Peripheral Olfactory Repertoire of the Lightbrown Apple Moth, *Epiphyas postvittana*. PLoS ONE.

[14] Qiu C.Z., Zhou Q.Z., Liu T.T., Fang S.M., Wang Y.-W., Fang X., Huang C.L., Yu Q.Y., Chen C.H., Zhang Z. (2018). Evidence of Peripheral Olfactory Impairment in the Domestic Silkworms: Insight from the Comparative Transcriptome and Population Genetics. BMC Genom..

[15] Adden A., Stewart T.C., Webb B., Heinze S. (2022). A Neural Model for Insect Steering Applied to Olfaction and Path Integration. Neural Comput..

[16] Rouyar A., Deisig N., Dupuy F., Limousin D., Wycke M.-A., Renou M., Anton S. (2015). Unexpected Plant Odor Responses in a Moth Pheromone System. Front. Physiol..

[17] Guo M.B., Du L.X., Chen Q.Y., Feng Y.L., Zhang J., Zhang X.X., Tian K., Cao S., Huang T.Y., Jacquin-Joly E. (2021). Odorant Receptors for Detecting Flowering Plant Cues Are Functionally Conserved across Moths and Butterflies. Mol. Biol. Evol..

[18] de Fouchier A., Walker W.B., Montagné N., Steiner C., Binyameen M., Schlyter F., Chertemps T., Maria A., François M.-C., Monsempes C. (2017). Functional Evolution of Lepidoptera Olfactory Receptors Revealed by Deorphanization of a Moth Repertoire. Nat. Commun..

[19] Hansson B.S., Stensmyr M.C. (2011). Evolution of Insect Olfaction. Neuron.

[20] Di C., Ning C., Huang L.Q., Wang C.Z. (2017). Design of Larval Chemical Attractants Based on Odorant Response Spectra of Odorant Receptors in the Cotton Bollworm. Insect Biochem. Mol. Biol..

[21] Tanaka K., Uda Y., Ono Y., Nakagawa T., Suwa M., Yamaoka R., Touhara K. (2009). Highly Selective Tuning of a Silkworm Olfactory Receptor to a Key Mulberry Leaf Volatile. Curr. Biol..

[22] Revadi S.V., Giannuzzi V.A., Rossi V., Hunger G.M., Conchou L., Rondoni G., Conti E., Anderson P., Walker W.B., Jacquin-Joly E. (2021). Stage-Specific Expression of an Odorant Receptor Underlies Olfactory Behavioral Plasticity in *Spodoptera littoralis* Larvae. BMC Biol..

[23] Zhang X.X., Liu Y., Guo M.B., Sun D.D., Zhang M.J., Chu X., Berg B.G., Wang G.R. (2024). A Female-Specific Odorant Receptor Mediates Oviposition Deterrence in the Moth *Helicoverpa armigera*. Curr. Biol..

[24] Itagaki H., Hildebrand J.G. (1990). Olfactory Interneurons in the Brain of the Larval Sphinx Moth *Manduca sexta*. J. Comp. Physiol. A.

[25] Zalucki M.P., Clarke A.R., Malcolm S.B. (2002). Ecology and Behavior of First Instar Larval Lepidoptera. Annu. Rev. Entomol..

[26] Tay W.T., Meagher R.L., Czepak C., Groot A.T. (2023). *Spodoptera frugiperda*: Ecology, Evolution, and Management Options of an Invasive Species. Annu. Rev. Entomol..

[27] Goergen G., Kumar P.L., Sankung S.B., Togola A., Tamò M. (2016). First Report of Outbreaks of the Fall Armyworm *Spodoptera frugiperda* (J E Smith) (Lepidoptera, Noctuidae), a New Alien Invasive Pest in West and Central Africa. PLoS ONE.

[28] Sun X.X., Hu C.X., Jia H.R., Wu Q.L., Shen X.J., Zhao S.Y., Jiang Y.Y., Wu K.M. (2021). Case Study on the First Immigration of Fall Armyworm, *Spodoptera frugiperda* Invading into China. J. Integr. Agric..

[29] WU Q.L., Jiang Y.Y., Hu G., Wu K.M. (2019). Analysis on Spring and Summer Migration Routes of Fall Armyworm (*Spodoptera frugiperda*) from Tropical and Southern Subtropical Zones of China. Plant Prot..

[30] Wu Q.L., Jiang Y.Y., Wu K.M. (2019). Analysis of Migration Routes of the Fall Armyworm *Spodoptera frugiperda* (J. E. Smith) from Myanmar to China. J. Plant Prot..

[31] Zhou Y., Wu Q.L., Zhang H.W., Wu K.M. (2021). Spread of Invasive Migratory Pest *Spodoptera frugiperda* and Management Practices throughout China. J. Integr. Agric..

[32] Cheruiyot D., Chiriboga Morales X., Chidawanyika F., Bruce T.J.A., Khan Z.R. (2021). Potential Roles of Selected Forage Grasses in Management of Fall Armyworm (*Spodoptera frugiperda*) through Companion Cropping. Entomol. Exp. Appl..

[33] Midega C.A.O., Pittchar J.O., Pickett J.A., Hailu G.W., Khan Z.R. (2018). A Climate-Adapted Push-Pull System Effectively Controls Fall Armyworm, *Spodoptera frugiperda* (J E Smith), in Maize in East Africa. Crop Prot..

[34] Scheidegger L., Niassy S., Midega C., Chiriboga X., Delabays N., Lefort F., Zürcher R., Hailu G., Khan Z., Subramanian S. (2021). The Role of *Desmodium intortum*, *Brachiaria* sp. and *Phaseolus vulgaris* in the Management of Fall Armyworm *Spodoptera frugiperda* (J. E. Smith) in Maize Cropping Systems in Africa. Pest Manag. Sci..

[35] Firake D.M., Behere G.T. (2020). Natural Mortality of Invasive Fall Armyworm, *Spodoptera frugiperda* (J. E. Smith) (Lepidoptera: Noctuidae) in Maize Agroecosystems of Northeast India. Biol. Control.

[36] Hussain A.G., Wennmann J.T., Goergen G., Bryon A., Ros V.I.D. (2021). Viruses of the Fall Armyworm *Spodoptera frugiperda*: A Review with Prospects for Biological Control. Viruses.

[37] Groot A.T., Dekker T., Heckel D.G. (2016). The Genetic Basis of Pheromone Evolution in Moths. Annu. Rev. Entomol..

[38] Zhang S., Liu F., Yang B., Liu Y., Wang G.R. (2023). Functional Characterization of Sex Pheromone Receptors in *Spodoptera frugiperda*, *S. exigua*, and *S. litura* Moths. Insect Sci..

[39] Zhang S., Jacquin-Joly E., Montagné N., Liu F., Liu Y., Wang G.R. (2023). Identification of an Odorant Receptor Responding to Sex Pheromones in *Spodoptera frugiperda* Extends the Novel Type-I PR Lineage in Moths. Insect Sci..

[40] Dong J.F., Yang H.B., Li D.X., Yu H.Q., Tian C.H. (2023). Identification and Expression Analysis of Chemosensory Receptors in the Tarsi of Fall Armyworm, *Spodoptera frugiperda* (J. E. Smith). Front. Physiol..

[41] Wang J.X., Wei Z.Q., Chen M.D., Yan Q., Zhang J., Dong S.L. (2023). Conserved Odorant Receptors Involved in Nonanal-Induced Female Attractive Behavior in Two *Spodoptera* Species. J. Agric. Food Chem..

[42] Sun Y.L., Jiang P.S., Dong B.X., Tian C.H., Dong J.F. (2022). Candidate Chemosensory Receptors in the Antennae and Maxillae of *Spodoptera frugiperda* (J. E. Smith) Larvae. Front. Physiol..

[43] Zhang L., Liu B., Zheng W.G., Liu C.H., Zhang D.D., Zhao S.Y., Li Z.Y., Xu P.J., Wilson K., Withers A. (2020). Genetic Structure and Insecticide Resistance Characteristics of Fall Armyworm Populations Invading China. Mol. Ecol. Resour..

[44] Gouin A., Bretaudeau A., Nam K., Gimenez S., Aury J.-M., Duvic B., Hilliou F., Durand N., Montagné N., Darboux I. (2017). Two Genomes of Highly Polyphagous Lepidopteran Pests (*Spodoptera frugiperda*, Noctuidae) with Different Host-Plant Ranges. Sci. Rep..

[45] Koutroumpa F.A., Monsempes C., François M.-C., Severac D., Montagné N., Meslin C., Jacquin-Joly E. (2021). Description of Chemosensory Genes in Unexplored Tissues of the Moth *Spodoptera littoralis*. Front. Ecol. Evol..

[46] Meslin C., Mainet P., Montagné N., Robin S., Legeai F., Bretaudeau A., Johnston J.S., Koutroumpa F., Persyn E., Monsempès C. (2022). *Spodoptera littoralis* Genome Mining Brings Insights on the Dynamic of Expansion of Gustatory Receptors in Polyphagous Noctuidae. G3 Genes Genomes Genet..

[47] Stamatakis A. (2014). RAxML Version 8: A Tool for Phylogenetic Analysis and Post-Analysis of Large Phylogenies. Bioinform. Oxf. Engl..

[48] Shimodaira H., Hasegawa M. (1999). Multiple Comparisons of Log-Likelihoods with Applications to Phylogenetic Inference. Mol. Biol. Evol..

[49] Livak K.J., Schmittgen T.D. (2001). Analysis of Relative Gene Expression Data Using Real-Time Quantitative PCR and the 2(-Delta Delta C(T)) Method. Methods.

[50] Liu Y.P., Zhang S., Cao S., Jacquin-Joly E., Zhou Q., Liu Y., Wang G.R. (2024). An Odorant Receptor Mediates the Avoidance of *Plutella xylostella* against Parasitoid. BMC Biol..

[51] Poivet E., Rharrabe K., Monsempes C., Glaser N., Rochat D., Renou M., Marion-Poll F., Jacquin-Joly E. (2012). The Use of the Sex Pheromone as an Evolutionary Solution to Food Source Selection in Caterpillars. Nat. Commun..

[52] Cao S., Liu Y., Guo M.B., Wang G.R. (2016). A Conserved Odorant Receptor Tuned to Floral Volatiles in Three Heliothinae Species. PLoS ONE.

[53] Liu X.H., Shi L.F., Khashaveh A., Shan S., Lv B.B., Gu S.H., Zhang Y.J. (2023). Loss of Binding Capabilities in an Ecologically Important Odorant Receptor of the Fall Armyworm, *Spodoptera frugiperda*, by a Single Point Mutation. J. Agric. Food Chem..

[54] Benton R., Sachse S., Michnick S.W., Vosshall L.B. (2006). Atypical Membrane Topology and Heteromeric Function of *Drosophila* Odorant Receptors in vivo. PLoS Biol..

[55] Legeai F., Malpel S., Montagné N., Monsempes C., Cousserans F., Merlin C., François M.-C., Maïbèche-Coisné M., Gavory F., Poulain J. (2011). An Expressed Sequence Tag Collection from the Male Antennae of the Noctuid Moth *Spodoptera littoralis*: A Resource for Olfactory and Pheromone Detection Research. BMC Genom..

[56] Krieger J., Raming K., Dewer Y.M.E., Bette S., Conzelmann S., Breer H. (2002). A Divergent Gene Family Encoding Candidate Olfactory Receptors of the Moth *Heliothis virescens*. Eur. J. Neurosci..

[57] Hoballah M.E., Turlings T.C.J. (2005). The Role of Fresh versus Old Leaf Damage in the Attraction of Parasitic Wasps to Herbivore-Induced Maize Volatiles. J. Chem. Ecol..

[58] Zakir A., Sadek M., Bengtsson M., Hansson B., Witzgall P. (2013). Herbivore-Induced Plant Volatiles Provide Associational Resistance against an Ovipositing Herbivore. J. Ecol..

[59] Binder B.F., Robbins J.C. (1997). Effect of Terpenoids and Related Compounds on the Oviposition Behavior of the European Corn Borer, *Ostrinia nubilalis* (Lepidoptera: Pyralidae). J. Agric. Food Chem..

[60] De Moraes C.M., Mescher M.C., Tumlinson J.H. (2001). Caterpillar-Induced Nocturnal Plant Volatiles Repel Conspecific Females. Nature.

[61] Konstantopoulou M.A., Krokos F.D., Mazomenos B.E. (2004). Chemical Composition of Corn Leaf Essential Oils and Their Role in the Oviposition Behavior of *Sesamia nonagrioide* Females. J. Chem. Ecol..

[62] Witzgall P., Ansebo L., Yang Z., Angeli G., Sauphanor B., Bengtsson M. (2005). Plant Volatiles Affect Oviposition by Codling Moths. Chemoecology.

[63] Carroll M.J., Schmelz E.A., Meagher R.L., Teal P.E.A. (2006). Attraction of *Spodoptera frugiperda* Larvae to Volatiles from Herbivore-Damaged Maize Seedlings. J. Chem. Ecol..

