# Supplementary material for: Functional Characterization of an Odorant Receptor Expressed in Newly Hatched Larvae of Fall Armyworm Spodoptera frugiperda
Source: Insects. 2024 Jul 26;15(8):564. doi: 10.3390/insects15080564 (PMC11354698; doi:10.3390/insects15080564)
Supplement: Supplementary file 1 [file insects-15-00564-s001.zip › insects-3086185-supplementary.pdf]

**Table S1.** Test compounds in this study.

| No. | odorant | CAS number | No. | odorant | CAS number |
|---|---|---|---|---|---|
| 1 | 2,6-Di-tert-butylphenol | 128-39-2 | 35 | Heptanal | 111-71-7 |
| 2 | Acetophenone | 98-86-2 | 36 | Phenylacetaldehyde | 122-78-1 |
| 3 | Eugenol | 97-53-0 | 37 | 4'-ethylacetophenone | 937-30-4 |
| 4 | 2-Phenylethanol | 60-12-8 | 38 | Jasmone | 488-10-8 |
| 5 | *trans*-3-Hexen-1ol | 928-97-2 | 39 | (±)-Camphor | 76-22-2 |
| 6 | 3,7-Dimethyl-3-octanol | 78-69-3 | 40 | 2-pentadecanone | 2345-28-0 |
| 7 | (1r)-(−)-Myrtenol | 19894-97-4 | 41 | (−)-Piperitone[1] | 4573-50-6 |
| 8 | (−)-*trans*-Pinocarveol | 547-61-5 | 42 | Myrcene | 123-35-3 |
| 9 | (−)-Linalool | 126-91-0 | 43 | (−)-*trans*-Caryophyllene oxide | 1139-30-6 |
| 10 | Linalool | 78-70-6 | 44 | Farnesene, mixture of isom | 502-61-4 |
| 11 | (+)-Cedrol | 77-53-2 | 45 | Ocimene | 13877-91-3 |
| 12 | *cis*-3-Hexen-1-ol | 928-96-1 | 46 | (R)-(+)-limonene | 5989-27-5 |
| 13 | 4-Methoxybenzyl alcohol | 105-13-5 | 47 | A-Pinene | 80-56-8 |
| 14 | 1-Octanol | 111-87-5 | 48 | (−)-β-Pinene | 18172-67-3 |
| 15 | Benzyl alcohol | 100-51-6 | 49 | Camphene | 79-92-5 |
| 16 | 4-hydroxy-4-methyl-2pentanone | 123-42-2 | 50 | A-Humulene[2] | 6753-98-6 |
| 17 | 1-octen-3-ol | 3391-86-4 | 51 | (S)-(−)-limonene | 5989-54-8 |
| 18 | Eucalyptol | 470-82-6 | 52 | A-Terpinene | 99-86-5 |
| 19 | B-citronellol | 106-22-9 | 53 | (−)-*trans*-Caryophyllene | 87-44-5 |
| 20 | Geraniol | 106-24-1 | 54 | Methyl benzoate | 93-58-3 |
| 21 | *cis*-2-hexen-1-ol | 928-94-9 | 55 | Decalactone | 705-86-2 |
| 22 | 1-heptanol | 111-70-6 | 56 | Geranyl acetate | 105-87-3 |
| 23 | 1s-(−)-verbenone | 1196-01-6 | 57 | (Z)-3-Hexenyl acetate | 3681-71-8 |
| 24 | 1-hexanol | 111-27-3 | 58 | Methyl 2-Methoxy benzoate | 606-45-1 |
| 25 | (S)-*cis*-verbenol | 18881-04-4 | 59 | Butyl salicylate | 2052-14-4 |
| 26 | *trans*-2-hexen-1-al | 6728-26-3 | 60 | Methyl phenylacetate | 101-41-7 |
| 27 | Salicylaldehyde | 90-02-8 | 61 | *trans*-2-Hexenyl acetate | 2497-18-9 |
| 28 | (±)-Citronellal | 106-23-0 | 62 | Benzyl acetate | 140-11-4 |
| 29 | Cinnamaldehyde | 104-55-2 | 63 | Methyl salicylate | 119-36-8 |
| 30 | 4-ethylbenzaldehyde | 4748-78-1 | 64 | 2-hexanol | 626-93-7 |
| 31 | 3-vinylbenzaldehyde | 19955-99-8 | 65 | 3-hexanol | 623-37-0 |
| 32 | Nonanal | 124-19-6 | 66 | *trans*-2-Hexen-1-ol | 928-95-0 |
| 33 | (1r)-(−)-myrtenal | 18486-69-6 | 67 | Octanal | 124-13-0 |
| 34 | Benzaldehyde | 100-52-7 | | | |

[1]. Purchased from TCI Shanghai. [2]. Purchased from J&K Scientific.

**Table S2.** Raw data of Functional Characterization of SfruOR40

| No. | odorant | Current Value(nA) | | | | | | |
|---|---|---|---|---|---|---|---|---|
| 1 | 2,6-Di-tert-butylphenol | 0 | 0 | 0 | 0 | 0 | 0 | 0 |
| 2 | Acetophenone | 0 | 0 | 0 | 0 | 0 | 0 | 0 |
| 3 | Eugenol | 0 | 0 | 0 | 0 | 0 | 0 | 0 |
| 4 | 2-phenylethanol | 0 | 0 | 0 | 0 | 0 | 0 | 0 |
| 5 | *trans*-3-Hexen-1ol | 0 | 0 | 0 | 0 | 0 | 0 | 0 |
| 6 | 3,7-Dimethyl-3-octanol | 0 | 0 | 0 | 0 | 0 | 0 | 0 |
| 7 | (1r)-(−)-myrtenol | 0 | 0 | 0 | 0 | 0 | 0 | 0 |
| 8 | (−)-trans-Pinocarveol | 0 | 0 | 0 | 0 | 0 | 0 | 0 |
| 9 | (−)-Linalool | 0 | 0 | 0 | 0 | 0 | 0 | 0 |
| 10 | Linalool | 0 | 0 | 0 | 0 | 0 | 0 | 0 |
| 11 | (+)-Cedrol | 0 | 0 | 0 | 0 | 0 | 0 | 0 |
| 12 | *cis*-3-hexen-1-ol | 0 | 0 | 0 | 0 | 0 | 0 | 0 |
| 13 | 4-Methoxybenzyl alcohol | 0 | 0 | 0 | 0 | 0 | 0 | 0 |
| 14 | 1-octanol | 0 | 0 | 0 | 0 | 0 | 0 | 0 |
| 15 | Benzyl alchol | 0 | 0 | 0 | 0 | 0 | 0 | 0 |
| 16 | 4-hydroxy-4-methyl-2pentanone | 0 | 0 | 0 | 0 | 0 | 0 | 0 |
| 17 | 1-octen-3-ol | 0 | 0 | 0 | 0 | 0 | 0 | 0 |
| 18 | Eucalyptol | 0 | 0 | 0 | 0 | 0 | 0 | 0 |
| 19 | B-Citronellol | 0 | 0 | 0 | 0 | 0 | 0 | 0 |
| 20 | Geraniol | 0 | 0 | 0 | 0 | 0 | 0 | 0 |
| 21 | *cis*-2-hexen-1-ol | 0 | 0 | 0 | 0 | 0 | 0 | 0 |
| 22 | 1-heptanol | 0 | 0 | 0 | 0 | 0 | 0 | 0 |
| 23 | 1s-(−)-verbenone | 0 | 0 | 0 | 0 | 0 | 0 | 0 |
| 24 | 1-hexanol | 0 | 0 | 0 | 0 | 0 | 0 | 0 |
| 25 | (S)-*cis*-Verbenol | 0 | 0 | 0 | 0 | 0 | 0 | 0 |
| 26 | *trans*-2-Hexen-1-al | 0 | 0 | 0 | 0 | 0 | 0 | 0 |
| 27 | Salicylaldehyde | 0 | 0 | 0 | 0 | 0 | 0 | 0 |
| 28 | (±)-Citronellal | 0 | 0 | 0 | 0 | 0 | 0 | 0 |
| 29 | Cinnamaldehyde | 0 | 0 | 0 | 0 | 0 | 0 | 0 |
| 30 | 4-ethylbenzaldehyde | 0 | 0 | 0 | 0 | 0 | 0 | 0 |
| 31 | 3-vinylbenzaldehyde | 0 | 0 | 0 | 0 | 0 | 0 | 0 |
| 32 | Nonanal | 0 | 0 | 0 | 0 | 0 | 0 | 0 |
| 33 | (1r)-(−)-myrtenal | 0 | 0 | 0 | 0 | 0 | 0 | 0 |
| 34 | Benzaldehyde | 0 | 0 | 0 | 0 | 0 | 0 | 0 |
| 35 | Heptanal | 0 | 0 | 0 | 0 | 0 | 0 | 0 |
| 36 | Phenylacetaldehyde | 0 | 0 | 0 | 0 | 0 | 0 | 0 |
| 37 | 4'-ethylacetophenone | 0 | 0 | 0 | 0 | 0 | 0 | 0 |
| 38 | Jasmone | 0 | 0 | 0 | 0 | 0 | 0 | 0 |
| 39 | (±)-Camphor | 0 | 0 | 0 | 0 | 0 | 0 | 0 |
| 40 | 2-pentadecanone | 0 | 0 | 0 | 0 | 0 | 0 | 0 |
| 41 | (−)-Piperitone | 0 | 0 | 0 | 0 | 0 | 0 | 0 |

| | | | | | | | | |
|---|---|---|---|---|---|---|---|---|
| 42 | Myrcene | 0 | 0 | 0 | 0 | 0 | 0 | 0 |
| 43 | (−)-trans-Caryophyllene oxide | 330 | 40 | 40 | 30 | 40 | 20 | 130 |
| 44 | Farnesene， mixture of isom | 0 | 0 | 0 | 0 | 0 | 0 | 0 |
| 45 | Ocimene | 0 | 0 | 0 | 0 | 0 | 0 | 0 |
| 46 | (R)-(+)-limonene | 0 | 0 | 0 | 0 | 0 | 0 | 0 |
| 47 | A-Pinene | 0 | 0 | 0 | 0 | 0 | 0 | 0 |
| 48 | (−)-β-Pinene | 0 | 0 | 0 | 0 | 0 | 0 | 0 |
| 49 | Camphene | 0 | 0 | 0 | 0 | 0 | 0 | 0 |
| 50 | A-Humulene | 600 | 80 | 70 | 140 | 90 | 70 | 390 |
| 51 | (S)-(−)-limonene | 0 | 0 | 0 | 0 | 0 | 0 | 0 |
| 52 | A-Terpinene | 0 | 0 | 0 | 0 | 0 | 0 | 0 |
| 53 | (−)-trans-Caryophyllene | 520 | 40 | 40 | 70 | 50 | 10 | 50 |
| 54 | Methyl benzoate | 0 | 0 | 0 | 0 | 0 | 0 | 0 |
| 55 | Decalactone | 0 | 0 | 0 | 0 | 0 | 0 | 0 |
| 56 | Geranyl acetate | 0 | 0 | 0 | 0 | 0 | 0 | 0 |
| 57 | (Z)-3-Hexenyl acetate | 0 | 0 | 0 | 0 | 0 | 0 | 0 |
| 58 | Methyl 2-Methoxy benzoate | 0 | 0 | 0 | 0 | 0 | 0 | 0 |
| 59 | Butyl salicylate | 0 | 0 | 0 | 0 | 0 | 0 | 0 |
| 60 | Methyl phenylacetate | 0 | 0 | 0 | 0 | 0 | 0 | 0 |
| 61 | *trans*-2-Hexenyl acetate | 0 | 0 | 0 | 0 | 0 | 0 | 0 |
| 62 | Benzyl acetate | 0 | 0 | 0 | 0 | 0 | 0 | 0 |
| 63 | Methyl salicylate | 0 | 0 | 0 | 0 | 0 | 0 | 0 |
| 64 | 2-hexanol | 0 | 0 | 0 | 0 | 0 | 0 | 0 |
| 65 | 3-hexanol | 0 | 0 | 0 | 0 | 0 | 0 | 0 |
| 66 | *trans*-2-Hexen-1-ol | 0 | 0 | 0 | 0 | 0 | 0 | 0 |
| 67 | Octanal | 0 | 0 | 0 | 0 | 0 | 0 | 0 |
